# Industrial Hemp (*Cannabis sativa* L.) Inflorescences as Novel Food: The Effect of Different Agronomical Practices on Chemical Profile

**DOI:** 10.3390/foods11223658

**Published:** 2022-11-16

**Authors:** Mattia Spano, Giacomo Di Matteo, Cinzia Ingallina, Anatoly Petrovich Sobolev, Anna Maria Giusti, Giuliana Vinci, Silvia Cammarone, Carola Tortora, Lara Lamelza, Sabrina Antonia Prencipe, Laura Gobbi, Bruno Botta, Federico Marini, Enio Campiglia, Luisa Mannina

**Affiliations:** 1 Department of Chemistry and Technology of Drugs, Sapienza University of Rome, Piazzale Aldo Moro 5, 00185 Rome, Italy; 2 Magnetic Resonance Laboratory “Segre-Capitani”, Institute for Biological Systems, CNR, Via Salaria Km 29.300, 00015 Monterotondo, Italy; 3 Department of Experimental Medicine, Sapienza University of Rome, Piazzale Aldo Moro 5, 00185 Rome, Italy; 4 Department of Management, Sapienza University of Rome, Via del Castro Laurenziano 9, 00161 Rome, Italy; 5 Department of Chemistry, Sapienza University of Rome, Piazzale Aldo Moro 5, 00185 Rome, Italy; 6 Department of Agricultural and Forest Sciences, University of Tuscia, Via San Camillo de Lellis Snc, 01100 Viterbo, Italy

**Keywords:** industrial hemp, agronomical practices, fertilization, harvesting, chemical profile

## Abstract

In this study, the effect of several agronomical practices on the chemical composition of hemp inflorescences, a potential novel food that needs to be further studied, was observed. Here, the case study of inflorescences from Ferimon cultivars is discussed and submitted to different agronomical practices (irrigation and fertilizers) in different years, and the inflorescences harvested in different periods were analyzed by a multimethodological approach. Targeted and untargeted methodologies allowed cannabinoids, total phenolic content, metabolite profile and antioxidant activity to be determined. The biomass and inflorescence yields were also reported. The whole data set was submitted to ANOVA-simultaneous component analysis. The statistic results allowed us to observe that irrigation was responsible for the (–)-Δ9-tetrahydrocannabinol (THC) and cannabidiol (CBD) increment. THC, cannabichromene (CBC), cannabigerol (CBG), succinate, and fructose resulted as higher in full female flowering than in the period of seed maturity. On the other hand, nitrogen supplementation led to an increase of iso-leucine, valine, and threonine. The obtained results underlined both the potential food application of hemp inflorescences, due to the rich chemical profile, and the strong effect of agronomical practices, mainly irrigation and harvesting, on the qualitative and quantitative characteristics of its metabolite profile.

## 1. Introduction

Hemp (*Cannabis sativa* L.) is an herbaceous, annual plant of eastern Asian origin, whose cultivation was forbidden in many countries for a long time due to the presence of tetrahydrocannabinol (THC), a psychoactive secondary metabolite contained in different parts of the plant. Recently, the European Union reintroduced the cultivation of some genotypes of *Cannabis sativa* L., also called industrial hemp, with a THC content lower than 0.2% *w*/*w* for fiber, seed, and inflorescence production [1]. In 2016, the Italian government approved a new regulation (Law n°242/2016), following the European one, regarding industrial hemp production, commercialization, and utilization. Therefore, in the next few years, the cultivation of industrial hemp is expected to increase thanks to the positive regulatory framework and the new products obtainable from this crop. In fact, besides the traditional utilization, industrial hemp products could be employed in several fields such as in functional food and nutraceuticals sectors [2,3]. In this regard, a rising interest in hemp metabolites such as fatty acids, flavonoids, amino acids, terpenes, terpenophenolics, sugars, and hydrocarbons, biosynthesized in the epidermal glands or glandular hairs of aerial parts of the plant [4], has been observed. These metabolites are contained in large amounts in inflorescences [5,6] and leaves [7,8].

To date, the European Union does not allow to use hemp inflorescences as food or food ingredient due to the presence of cannabinoids. Cannabinoids are now included in the novel food catalogue, but for the final authorization [9], further assessment studies are required.

Therefore, the prospect of using hemp inflorescences as a novel food has prompted scientific research to deepen the knowledge of the chemical composition of the hemp inflorescences with a particular focus on classes of molecules important in the food-nutraceutical sector. In this context, several studies on hemp inflorescences have reported the presence of amino acids, sugars, organic acids, polyphenols, etc. [10,11,12,13].

Several factors such as different genotypes [14,15], climatic conditions, and agronomic practices [11] can influence the hemp inflorescences development and, consequently, the chemical profile. Therefore, it is extremely important to know the effects of different management practices on the physiological accumulation of secondary metabolites and on the inflorescence yield for the economic sustainability and for choosing the proper agronomic practices to obtain inflorescences suitable for food uses.

Several studies have been carried out on the effect of specific agronomical practices such as fertilization [16,17,18], irrigation [19,20], and harvesting period variation [11,13,21] on hemp cultivation.

Regarding the fertilization, nitrogen (N) and phosphorus (P) fertilizers play an important role in crop growth and development [22]. The knowledge of the proper mineral nutrient requirement of industrial hemp is important not only for inflorescence yield and quality, but also to reduce the risks of environmental pollution from runoff or leaching of excess nutrients [23]. In a recent study on the growth of marijuana genotypes conducted in a controlled environment, inflorescence yield responded quadratically to N and P, with the optimal concentrations in nutrient solution predicted to be 194 and 59 mg L^−1^, respectively [24]. Moreover, a negative correlation between cannabinoid content (THC and CBG) and increasing fertilizer rates [25,26] has been reported. However, information regarding N and P fertilization rates for inflorescence production in the open field is limited, and more research is needed to determine the proper use of fertilizers to obtain inflorescences with high yield and quality.

In the Mediterranean environment, another important agronomic factor for industrial hemp production is water availability, since during the cropping period, the climate is generally characterized by drought and high temperatures. Therefore, for the crop in a semi-arid Mediterranean environment, watering is often needed. Cosentino et al. [27] have suggested that irrigation water supply for industrial fiber hemp production ranges between 250 and 450 mm for early and late genotypes, respectively. However, it is feasible that the optimal water supply for irrigation is different for inflorescence production compared to fiber production. Low humidity conditions in open field have turned out to enhance the terpene content of essential oils [28]. The effect of irrigation on the cannabinoid content is still debated. Caplan et al. [29] have observed that drought-stressed hemp plants increased the concentrations and yield of major cannabinoids compared with a well-irrigated control, whereas other authors did not note clear responses of cannabinoid content in relation to the irrigation doses [30].

The harvesting period also plays a pivotal role in the chemical profile of inflorescences. Ingallina et al. [11] have found that, in monoecious industrial hemp cultivars, the content of proline drastically decreased after early flowering, whereas glucose, citrate and malate reached the highest content in the late flowering period. Similarly, in dioecious hemp cultivars, the chemical composition of inflorescences varies during the flowering period. Spano et al. [13] have observed that at the beginning of flowering, female inflorescence extracts have been characterized by a high content of sucrose and myo-inositol, whereas at the end of flowering, the highest levels of glucose, fructose, choline, trigonelline, malic acid, formic acid, and some amino acids were observed.

Therefore, up to now, the research has been focused on the study of individual agronomical practices and their effect on cannabinoid content. A more complete chemical profile of hemp inflorescences has been reported in a few studies where only the harvesting period effect has been investigated [11,13].

In this paper, the combined effect of nitrogen and phosphorus fertilizer, irrigation, harvesting time and cultivation year on inflorescence chemical profile was investigated considering as a case study the monoecious Ferimon cultivar. A multimethodological approach based on both targeted (UHPLC-DAD, UV-Vis spectrophotometry) and untargeted (NMR) methodologies and chemometric analysis was applied.

## 2. Materials and Methods

### 2.1. Chemicals

Gradient grade water, acetonitrile, chloroform, methanol, and formic acid were purchased from Merck Life Science (Milano, Italy). Cannabinoid reference standards dissolved in methanol, namely, cannabidivarin (CBDV, 1 mg/mL), cannabigerol (CBG, 1 mg/mL), cannabidiol (CBD, 1 mg/mL), cannabidiolic acid (CBDA, 1 mg/mL), cannabinol (CBN, 1 mg/mL), (–)-Δ9-tetrahydrocannabinol ((–)-Δ9-THC, 1 mg/mL), Δ9-tetrahydrocannabinolic acid ((–)-Δ9-THCA, 1 mg/mL), (–)-Δ8-tetrahydrocannabinol ((–)-Δ8-THC, 1 mg/mL), and cannabichromene (CBC, 1 mg/mL) with purity ≥ 99%, were purchased from Merck Life Science (Milano, Italy). Deuterated water (D_2_O) 99.97 atom% of deuterium and 3-(trimethylsilyl)-propionic-2,2,3,3-d_4_ acid sodium salt (TSP) were purchased from Euriso-Top (Saclay, France). Magnesium oxide (MgO), potassium phosphate monobasic (KH_2_PO_4_), and potassium phosphate dibasic (K_2_HPO_4_) 2,2-azino-bis (3-ethyl-benzothiazoline-6-sulfonic acid) diammonium salt (ABTS), 2,2-diphenyl-1-picrylhydrazyl (DPPH), Folin–Ciocalteu reagent, gallic acid (GA), and sodium carbonate (Na_2_CO_3_) were purchased from Merck Life Science (Milano, Italy).

### 2.2. Experimental Site and Weather Conditions

Field experiments were conducted during the 2017 and 2018 growing seasons in Central Italy at the Experimental Farm of the University of Tuscia in Viterbo (latitude 42°25′ N, longitude 12°04′ E and altitude 295 m above sea level). The soil is volcanic with the following main characteristics at 0–30 cm depth: 11.2% clay, 14.7% silt, 74.1% sand, pH 6.7 (water, 1:2.5), organic matter 1.2% (Lotti method), total N 0.93 g kg^−1^ of dry soil (Kjeldahl method), available P_2_O_5_ 41 mg kg^−1^, and exchangeable K_2_O 534 mg kg^−1^. The climate of the experimental area is typically Mediterranean, characterized by a dry and hot summer and humid and mild winter. Weather data (rainfall, maximum and minimum air temperatures, and potential vapor-transpiration) were recorded daily from an automatic meteorological station located 100 m from the experimental fields. Meteorological data are reported at 10-day intervals for the period in which the experiment was conducted (Supplementary Table S1). As expected, in both experimental years, the average air temperatures gradually increased from sowing (April) to the hemp reproductive period and reached the maximum values in the first decade of August. However, there were remarkable differences between the experimental years. The range of temperature between minimum and maximum values was generally larger in 2017 compared to 2018. In fact, the temperature throughout the experimental period ranged from 8 °C in May to 38 °C in August 2017, whereas the temperature in the same period ranged from 11 °C in May 2018 to 34 °C in August 2018 (Supplementary Table S1). As a consequence, the average temperature in 2017 was significantly higher compared to 2018, especially during the flowering period. Furthermore, the total rainfall from hemp emergence until full flowering was 109 mm in 2017 and 306 mm in 2018; with a particular concentration in the last ten days of July 2017 (48 mm), and May 2018 (150 mm). Similar values were observed for potential evapotranspiration in both cropping seasons (618 and 595 mm in 2017 and 2018, respectively). From the weather data it is evident that the 2017 was characterized by a longer period of drought compared to 2018 during the hemp growing period.

### 2.3. Field Experiment Description, Experimental Design, and Treatments

One week before hemp sowing, where foreseen, the plots were fertilized with phosphorus (P) at a dose of 45 or 90 kg P_2_O_5_ ha^−1^ as triple superphosphate. After applying the fertilizer, the soil was disked twice for seedbed preparation. The hemp variety used in this study was Ferimon, a French monoecious cultivar listed in the EU database of registered hemp varieties and commonly used for seed production, that also showed good performance for inflorescence yield in the Mediterranean environment of Central Italy [31]. Industrial hemp was sown with an experimental sowing machine (Wintersteiger, Ried im Innkreis, Austria) on 26 April 2017 and 3 May 2018 planting the seeds in rows at 0.25 m interrow spacing. The hemp seed rate was 15 kg ha^−1^. In both years, in order to avoid emergence failure, the field was irrigated just after sowing by sprinkler irrigation. Ten days after emergence, the hemp seedlings were thinned manually in order to reach the density of 80 plants m^−2^, in accordance with the density used in a similar environment in recent research on hemp cultivation [31]. Where foreseen, nitrogen (N) fertilization was applied at a ratio of 75 or 150 kg N ha^−1^ as ammonium nitrate, split into three doses applied at 15, 30 and 45 days after crop emergence (33% of the total amount for each application). Following each N fertilization, a manual hoe was applied to incorporate the N fertilizer and to control inter-row weeds. Potassium fertilizer was not applied due to the high levels present in the soil. Irrigation water was supplied in the irrigated plots by means of drip irrigation placing on the soil surface one pipeline per row immediately after hemp seedling thinning. The amount of water input was determined by evapotranspiration estimated by class A pan evaporimeter and converted by crop coefficients, returning 90% of the evapotranspirated water from the crop. The hemp inflorescences were harvested twice: at full female flowering stage (A), and at seed maturity stage (B). In 2017, the following experimental treatments were applied in factorial combination: (i) one irrigation regime (irrigated crop); (ii) two doses of phosphorus (0 and 90 kg P_2_O_5_ ha^−1^); (iii) two doses of N fertilization (0 and 150 kg of N ha^−1^). In 2018, further variables were considered, by applying the following treatments: (i) two irrigation regimes (irrigated and not-irrigated crop); (ii) three doses of phosphorus (0, 45 and 90 kg P_2_O_5_ ha^−1^); and (iii) three doses of N fertilization (0, 75 and 150 kg of N ha^−1^), Supplementary Figure S1.

In 2017, the experimental design of the field was a split-plot, where the main plots were represented by the phosphorus fertilization, and the sub-plots were the N fertilization. In 2018, the experimental design of the field was a split-split-plot, where the main plots were represented by the irrigation regimes, the sub-plots were the phosphorus fertilization, and the sub-sub-plots were the N fertilization. The main plot size was 135 m^2^ (9 m × 15 m), the subplot size was 67.5 m^2^ (4.5 m × 15 m), and the sub-sub-plot size was 22.5 m^2^ (4.5 m × 5 m). In both years, the main plots in the field trial were separated by 8 m wide alleys for equipment operation.

### 2.4. Field Measurements and Data Collection

Immediately after hemp seedling thinning, a 2.0 m central row was randomly selected to carry out phenological observations, that were performed daily when the flowering stage was approaching. Two inflorescence harvests were carried out during the hemp reproductive period: the first one was carried out at full female flowering (50% of the bracts formed), encoded as phase 2302, whereas the second harvest occurred at seed maturity (50% of seeds hard), encoded as phase 2306 [32]. At each harvesting time, the plants in the 4 middle rows of each sub-sub-plot, corresponding at 1 m^2^ area, were manually cut just above the soil surface. The aboveground biomass was split into two fractions: stems and inflorescence by cutting the upper part of the plant stem, and the stem ramifications when present, at the insertion level of the first basal flower of the inflorescence. Both flowers and floral leaves were included in the inflorescence for determining the chemical profile, whereas the seeds present at the second inflorescence harvest were manually separated. The fresh material was immediately weighed, and a sub-sample was oven dried at 70 °C until constant weight to determine the dry matter content of stems, inflorescences, and seeds when present. The remaining part of the inflorescences were pulverized with liquid nitrogen by using mortar and pestle and then was preserved at a temperature of −80 °C until subsequent laboratory analyses.

In order to have information relevant for the economic sustainability, the biomass and inflorescences yield, inflorescences/biomass ratio (inf/bio) have been measured and reported.

### 2.5. UHPLC Analysis for Cannabinoid Profile

Fresh inflorescences were dried at 70 °C for 48 h before extraction. After drying, 100 mg of inflorescences were extracted with 4 mL of methanol in an ultrasonic bath for 30 min. The residual pellet was then extracted other two times. For each extract, an aliquot of 1 mL was filtered with a 0.45 μm PTFE membrane and finally analyzed.

Analyses were carried out by using an Ultimate 3000 ultra-high-performance liquid chromatography (UHPLC) (Thermo Fisher Scientific, Rodano, Italy), with a binary gradient system, an automatic injector, a thermostatic column compartment, and a diode array detector. The system was controlled by Chromeleon Chromatography Data System software (Thermo Fisher Scientific, 1.0.5. v, Waltham, MA, USA, 2018). All separations were performed by using a Titan C 18 column (10 cm × 3 cm, 1.9 μm, Sigma Aldrich (Milan, Italy)) with a mobile phase composed of 0.1% formic acid in water (A) and 0.1% formic acid in acetonitrile (B). The used elution conditions were the same previously described [13].

Cannabinoid concentrations were expressed as % *w*/*w* (g for 100 g of dried inflorescence) ± SD (standard deviation). Three replications were made for each sample.

### 2.6. Untargeted NMR Analysis

For the untargeted NMR analysis, Bligh–Dyer extraction procedure was applied, following the previously described conditions [33]. The hydroalcoholic extract was dried and then solubilized with 0.75 mL of 400 mM phosphate buffer/D_2_O, containing a 1 mM 3-(trimethylsilyl)-propionic-2,2,3,3-d_4_ acid sodium salt (TSP) as an internal standard, and then transferred into a 5 mm NMR tube. NMR analyses were carried out on a Bruker AVANCE 600 spectrometer operating at the proton frequency of 600.13 MHz and equipped with a Bruker multinuclear z-gradient 5 mm probe head (Milan, Italy). ^1^H spectra were acquired at 28 °C using the following parameters: 256 transients, suppression of residual water signal (HDO) using a pre-saturation, 5 s recycle delay, 45° pulse of 7.5 μs, and 32 K data points. Metabolites identification, see Supplementary Table S2, was carried out based on the NMR results previously reported using the same experimental condition [11].

### 2.7. Spectrophotometric Analysis of Total Phenolics Content and Antioxidant Activity

The extraction of phenolic compounds was carried out as follows: 0.5 g of fresh hemp inflorescences were added to 2.5 mL of MeOH/H_2_O 7:3 *v*/*v* solution. Samples were then shaken for 1 min, sonicated in an ultrasonic bath for 5 min, and finally centrifuged for 10 min at 2500× *g*. After supernatant removal, the residual pellet was extracted again using the same conditions. Both obtained supernatants were united to a final volume of 5 mL.

Total phenolics content (TPC) was determined using the Folin–Ciocâlteu method, optimized in a previous work for hemp inflorescences [13]. TPC was expressed as milligrams of gallic acid equivalents (GAE) kg^−1^: a calibration curve was created, with a concentration range from 15 to 500 mg/L (R^2^ = 0.9925). The measurements were carried out in triplicate for each sample and reported as mean value ± SD.

The antioxidant activity of hemp hydroalcoholic extracts was determined by applying DPPH and TEAC assays. The free radical scavenging activity of fresh hemp inflorescence extracts was evaluated by means of DPPH assay, according to a previously reported method [34]. The scavenging activity was measured at 517 nm. Values were reported as EC_50_ value ± SD; EC_50_, corresponded to the extracts’ concentration providing 50% of radicals scavenging activity. Gallic acid dissolved in methanol, ranged between 1 and 100 mg/L, was used as reference compound.

The Trolox equivalent antioxidant capacity (TEAC) of hemp inflorescence extracts was estimated by the ABTS radical scavenging assay, according to Omono et al. [35], with some modifications. All measurements were performed by adding 3.6 mL of the ABTS radical solution to 0.4 mL of hemp extracts, leaving it in darkness for 15 min. The decolorization of ABTS●* was evaluated by measuring the absorbance at 734 nm, and then expressed as milligrams Trolox Equivalent (TE) per g of hemp inflorescence (mg TE/g), obtained by a calibration curve ranging from 0.5 μM TE to 200 μM TE (R^2^ = 0.9963).

The assays were carried out in triplicate for each sample and reported as mean value ± SD.

### 2.8. Statistical Analysis

To evaluate the effect of the different cultivation conditions on the total defined chemical profile of hemp inflorescences, ANOVA-simultaneous component analysis (ASCA) was used. ASCA [36] is a multivariate method specifically proposed to analyze multivariate data coming from designed experiments and couples a multivariate ANOVA decomposition, to disentangle the contribution of each design term on the multivariate profile, with Simultaneous Component Analysis (SCA), that under the ANOVA constraints boils down to Principal Component Analysis (PCA), for the chemical interpretation of the observed effects.

If a term is found to be statistically significant, interpretation of the observed effect in terms of the multivariate profile recorded is made by SCA of the corresponding effect matrix. As explained above, under the constraints of ANOVA, the use of SCA is equivalent to a standard PCA on the matrix.

Since the effect matrices are only made of the mean profiles of the different factor/interaction levels, for the sake of a better interpretation, it is common practice, after the SCA model is calculated, to also project onto the resulting component the residuals, so as to have an idea of the expected within-level variability [37].

The obtained results, combined with the application of chemometric analysis, allowed us to observe the effect of the considered agronomical variables (nitrogen and phosphorus fertilization, irrigation, harvesting period) on the quali-quantitative features of the Ferimon metabolite profile.

## 3. Results and Discussion

### 3.1. Chemical Characterization

The chemical profile of Ferimon inflorescences was investigated by applying both targeted (UHPLC-DAD, UV-Vis spectrophotometry) analyses focused on cannabinoids and total phenols determination and NMR untargeted methodology to obtain the metabolite profile. The results will be discussed separately.

Cannabinoids. The UHPLC-DAD analysis of *Cannabis sativa* L. methanolic extracts allowed us to quantify Δ9-THC, CBD, Δ9-THCA, CBDA, CBG, and CBC. The concentrations, expressed as % *w*/*w* of dry matter ± SD, are reported in Table 1.

All the analyzed samples were characterized by a THC content below the legal limit, whereas CBD turned out to be the most abundant cannabinoid. In a previous study [11], the inflorescences of Ferimon hemp cultivar grown in 2016 in Central Lazio with different agronomical practices (irrigation and 100 kg ha^−1^ of N) have shown a different cannabinoids profile with the presence of CBDV and CBN not detected here. The cannabinoid profile of the Ferimon hemp cultivar grown in Slovenia in 2017 (agronomical practices not reported) [38] has shown a very high CBDA content, whereas CBD, THC, and CB contents were lower with respect to those here reported.

NMR metabolite profile. The NMR metabolomic profile of hydroalcoholic Bligh–Dyer inflorescence extracts was obtained using literature data [11] relative to Ferimon cultivar. Four sugars, four organic acids, eleven amino acids, choline, and trigonelline were detected and quantified in all the investigated samples. The value relative to each compound was reported on Supplementary Tables S3–S6.

Total phenolic content (TPC) and antioxidant activity. The TPC and the antioxidant activity of Ferimon inflorescence were determined by means of DPPH and ABTS assays, see Table 2 for samples relative to 2017 and 2018. In the previously study [11], the TPC of Ferimon cultivar showed a value lower than the one here reported. Moreover, another study has analyzed the antioxidant activity of Ferimon cultivar cultivated in Central Italy [39] (irrigation and fertilization practices not reported) and harvested during the flowering stage; anyway, since the results have been expressed using another unit of measurement, a comparison with the reported results was not possible.

To take into account the whole set of data, ASCA analysis was carried out at first on data matrices relative to 2017 and 2018 separately, and successively on the joint data. The comparison between continuous irrigation and absence of irrigation was carried out only in 2018.

### 3.2. The 2017 Year-Data

The 2017 year-data, relative only to irrigated fields, were acquired as a result of three controlled factors, each factor at two levels: harvesting period ((female flowering stage (A) and seed maturity stage (B)), P dose (0 and 90 kg P_2_O_5_ ha^−1^), and N dose (0 and 150 kg of N ha^−1^). ASCA applied to the matrix of experimental data, underlined that the largest part of the variance was due to the effect of harvesting (40.37%), followed by N dose (10.08%) and P dose (6.61%) factors. Permutation testing used to evaluate the significance of each of the design terms (main effects and interactions) showed that only the effect of harvesting period and N were statistically significant (*p* < 0.05). However, the effect of P fertilization was only slightly above the significance threshold (*p* = 0.0558). Accordingly, only the effect matrices for harvesting time and N fertilization were subjected to the successive SCA analysis.

**Figure 1:**
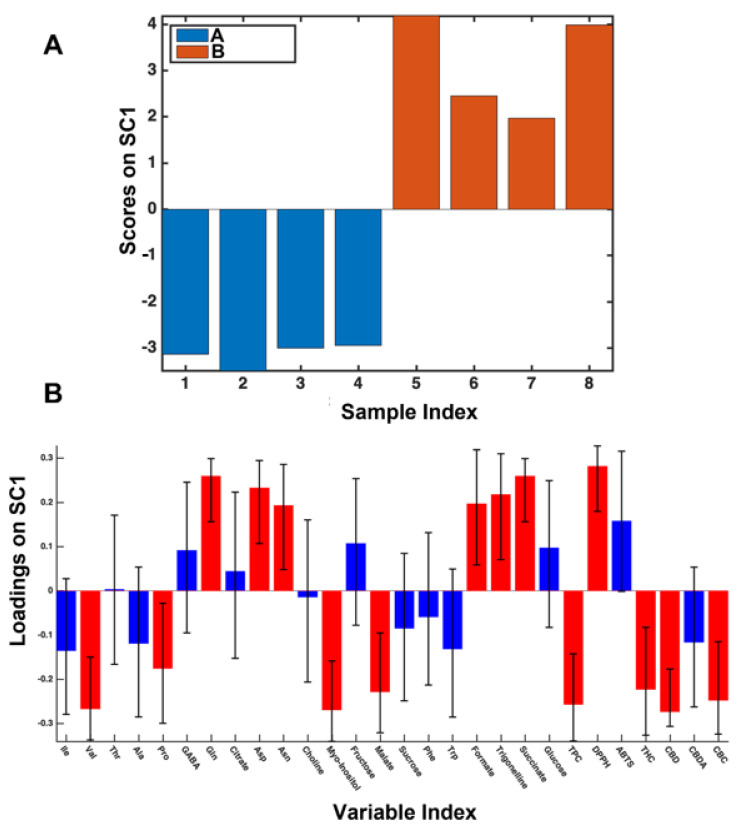
Scores (**A**) and loadings (**B**) plots of SC1 component extracted from SCA analysis regarding the harvesting effect on 2017 samples.

Harvesting. The harvesting period was the agronomical factor that mainly affected the chemical variability of the analyzed samples with stage A being characterized by higher levels of Val (Valine), Pro (Proline), Myo-inositol, Malate, TPC, THC, and CBC, whereas stage B samples were richer in Gln (Glutamine), Asp (Aspartate), Asn (Asparagine), Formate, Trigonelline, Succinate, and DPPH, see scores and loadings of ASCA displayed on Figure 1.

The scores plot on the SC component shows that stages A and B correspond to negative and positive scores, respectively. This information is fundamental to interpret the corresponding loadings plot, where the variables that significantly contribute to the component are highlighted as red bars. Indeed, all the variables with positive loadings are higher in stage B samples, whereas the ones with negative loadings are higher in stage A.

Moreover, an increase of biomass and inflorescences yield was observed from stage A to stage B, Table 3.

**Figure 2:**
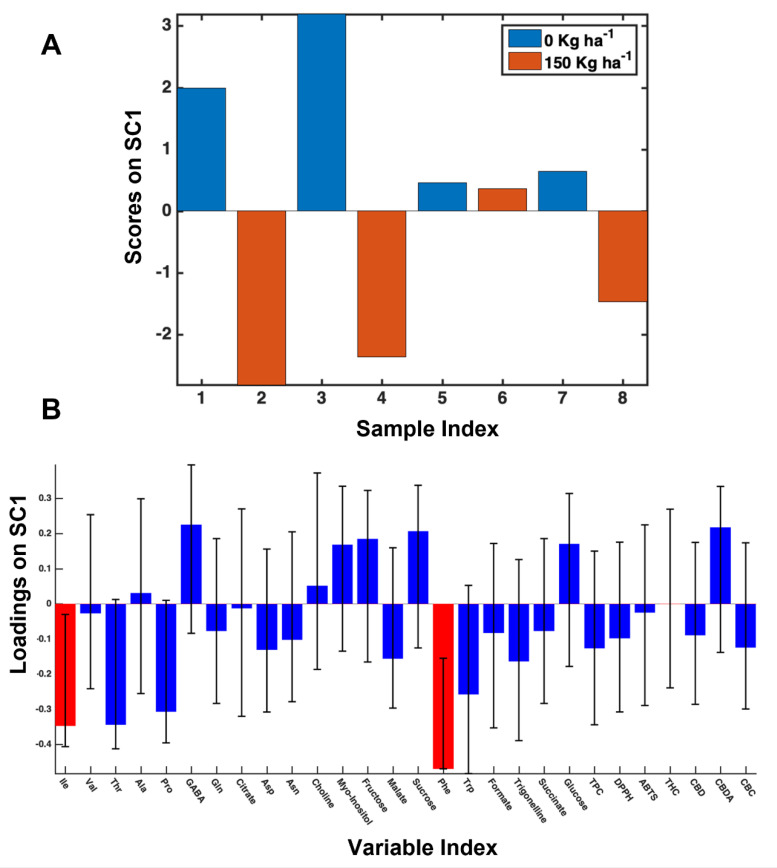
Scores (**A**) and loadings (**B**) plots of SC1 component extracted from SCA analysis regarding the N fertilization effect on 2017 samples.

Fertilization. The 150 kg ha^−1^ dose of N caused an increase of several amino acids, namely Ile (Isoleucine), Phe (Phenylalanine), Thr (Threonine), Pro, and Trp (Tryptophan). Among these, Ile and Phe were the most statistically significant increased, as shown by scores and loadings plot of the SC component extracted from the SCA analysis regarding the effect of N fertilization, Figure 2. In particular, the scores plot highlights that levels of 0 kg ha^−1^ and 150 kg ha^−1^ correspond to positive and negative scores, respectively. On the other hand, in the loadings plot, the red error bars indicate the 95% confidence interval of the coefficients, estimated by bootstrapping. On the contrary, no statistically significant effect of 0 kg ha^−1^ N were observed, although an increase of GABA (γ-aminobutyrate), Sucrose, and CBDA was observed.

Note that high N dosed caused an increase of both biomass and inflorescences yield, see Table 3.

### 3.3. The 2018 Year-Data

The 2018 year-data were acquired as a result of four controlled factors: P dose (0, 45 and 90 kg P_2_O_5_ ha^−1^), N dose (0, 75 and 150 kg of N ha^−1^), irrigation (irrigated or not), and harvesting period (female flowering stage (A) and seed maturity stage (B)). ASCA applied to the matrix of experimental data allowed us to observe that the largest part of the variance was due to the effect of irrigation (23.95%), followed by harvesting (21.76%), N fertilization (4.27%) and P dose (3.14%). On the other hand, permutation testing evidenced that all the design terms (main effects and interactions) are statistically significant (*p* < 0.05).

Harvesting effect. An increase of Choline, Myo-inositol, Fructose, Sucrose, Succinate, THCA, CBC, and CBG was observed in stage A, whereas stage B was characterized by an increase of Gln, Asp, Asn, Malate, Trp, Formate, TPC, CBD, and CBDA, as obtained by SCA.

Irrigation. Irrigation turned out to have the highest influence on hemp inflorescences chemical variability. When continuous irrigation was applied, an increase of DPPH, THC, and CBC was observed as underlined by SCA. It is noteworthy that DPPH increment was more appreciable in stage B. On the contrary, from SCA analysis, it emerged that the absence of irrigation caused an increase of several metabolites such as Val, Ala (Alanine), GABA, citrate, Asn, choline, fructose, malate, sucrose, formate, trigonelline, succinate, and glucose.

Additionally, the irrigation practice strongly affected the biomass and inflorescences yield. The absence of irrigation caused a strong stress condition in plants, thus reducing biomass and inflorescences at least of three times, see Table 3. It is noteworthy that in both irrigation and absence of irrigation conditions, the inf/bio ratio remained the same. This behavior has also been previously observed in Ermes and Carma cultivars grown in Spain, where a reduction of irrigation caused a decrease of biomass yield [19].

**Figure 3:**
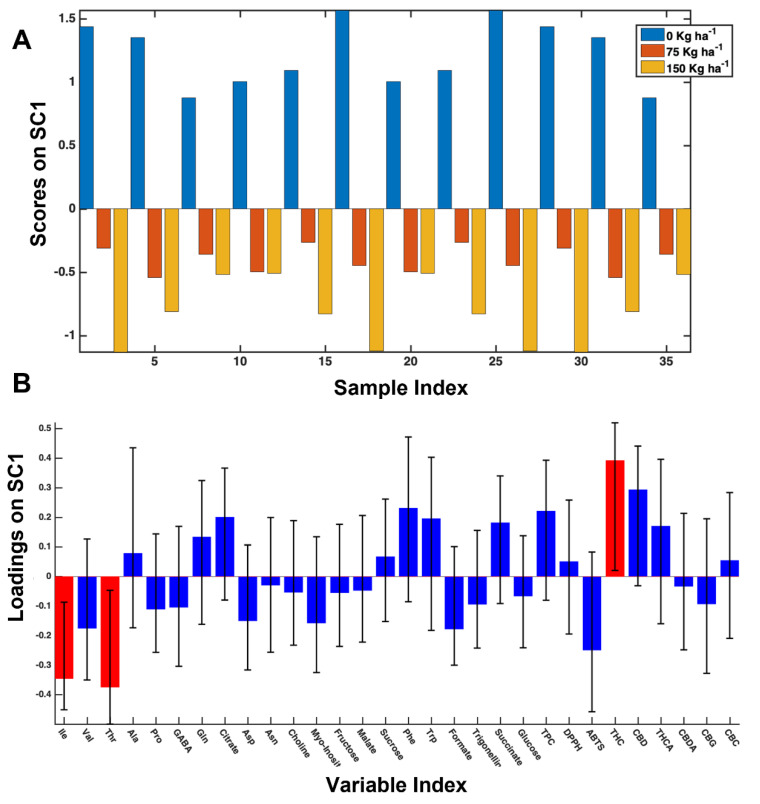
Scores (**A**) and loadings (**B**) plots of SC1 component extracted from SCA analysis regarding the N fertilization effect on 2018 samples.

Fertilization. The inspection of the scores plot (Figure 3A) indicates a clear effect of nitrogen supplementation, whereas at the same time little difference in increasing the N level from 75 to 150 kg ha^−1^ is observed. When this fertilizer was applied, a significant increment of Ile and Thr was observed. Additionally, Val, Pro, GABA, Asp, Myo-inositol, Formate, and GABA also increased, as shown by scores and loadings plot of the SC component extracted from the SCA. On the contrary, high supplementation of N caused a decrease of some cannabinoids, namely THC, THCA, and CBD, and also Phe, Trp, succinate, and TPC. Among these metabolites, THC was subjected to the most statistically significant decrease, see Figure 3. The obtained results allowed us to observe how N fertilization mainly affects the amino acids content, with most of them increasing when it was supplied. Moreover, the use of 75 kg ha^−1^ had the same effect of the higher 150 kg ha^−1^ dose, thus giving helpful information that can be used for reducing fertilizer waste [40].

Although P fertilization did not appear to have a significant influence on the inflorescences chemical profile, an increase of Phe was observed when this fertilizer was used.

N fertilization strongly affected hemp yield, with the behavior being connected with the presence or the absence of irrigation. In particular, in irrigated plants, the use of high N doses caused an increase of both biomass and inflorescences yield. These data were confirmed by literature data where high dosages of N have caused an increase of hemp biomass yield [16,17,18]. Moreover, a synergistic effect of combination of N fertilization and irrigation on hemp biomass yield was observed, with the highest yield when the highest dosage of N (150 kg ha^−1^) was used. On the contrary, when no irrigation was supplied, the use of high N doses caused a reduction of biomass and inflorescences yield, since high N dosages enhance the plant stress condition in not-irrigated plants. Similarly to what was observed for the chemical profile variations, P fertilization did not appear to influence the biomass and inflorescences yield.

### 3.4. 2017- and 2018-Years Data Set

The cultivation year represents an important factor that can strongly affect the chemical profile of food and vegetable matrices. Additionally, in this case, comparing the data obtained in 2017 and 2018, it was possible to observe a clear change of the chemical profile of hemp inflorescences. Interestingly some differences were observed in the cannabinoids profile, with THCA and CBG not being detected in inflorescences grown in 2017. The relevant effect of cultivation year was demonstrated by ASCA carried out on the joint data, by considering four factors: P dose (0 and 90 kg P_2_O_5_ ha^−1^), N dose (0 and 150 kg of N ha^−1^), harvesting (A and B) and year (2017 and 2018). All data were referred to irrigated crops. ASCA applied to the matrix of experimental data underlined that the largest part of the variance was due to the effect of year (48.59%), followed by that of harvesting (14.16%), N dose (3.79%), and P dose (2.08%). On the other hand, permutation testing evidenced that all the design terms (main effects and interactions) are statistically significant (*p* < 0.05).

The SCA regarding the effect matrix for the two years shows that almost all the metabolites were found to be present at significantly higher levels in 2018. The only exceptions were TPC, CBDA, and DPPH values, which higher in 2017, whereas Pro, Gln, Asp, Asn, Malate, CBD, THCA, and ABTS values did not show any significant difference.

Comparing the results obtained in 2017 and 2018, it was possible to observe that some metabolites such as Gln, Asp, Asn, and formate increased their content during the harvesting period in both years in agreement with data previously reported for the Ferimon cultivar [11].

Regarding yield data, only a slight increase of biomass and inflorescence was observed in 2018 with respect to 2017. In both 2017 and 2018, harvesting stage B was characterized by higher values of biomass and inflorescences yield, with a major value of inflorescences/biomass (inf/bio) ratio with respect to those calculated in stage A. This data suggested that the biomass increase observed in stage B was mainly due to a major inflorescences production, as also demonstrated by the inf/bio increase.

The reported results underlined that the agronomical practices as well as the cultivation year can strongly influence the chemical profile and the yield of hemp inflorescences. In particular, cultivation year and harvesting period and N fertilization significantly affected the inflorescences chemical profile. It is also important to note that irrigation and harvesting during the full female flowering period cause an increase of THC and CBD (THC content always below the legal limit). On the other hand, the absence of irrigation caused an increase of several amino acids, sugars, and organic acids, as well as a reduction of biomass and inflorescences yield, by triggering a stress condition in the plant. Regarding the harvesting period, some metabolites such as glutamate, asparagine, aspartate, and formate increased during the time in both 2017 and 2018, as observed previously [13]. Among fertilization, nitrogen supplementation had a major influence on inflorescence chemical variation with respect to phosphorus by affecting the content of amino acids and cannabinoids. Moreover, nitrogen fertilization strongly affected the biomass and inflorescences yield with the behavior being strictly related to the presence or absence of irrigation. In particular, when irrigation is applied, the use of high nitrogen dosages causes important yields to increase.

Up to now, the studies on the inflorescences reported in the literature are often not comparable since they were carried out in different conditions or more simply since the agronomical conditions are not reported. For this reason, it is important to improve the research in this field since, as demonstrated in this study, the agronomic practices can influence the chemical composition of hemp inflorescences. This issue can be firstly a notable factor if, for instance, THC concentration is considered. Indeed, its amount has to be below the legal limits to avoid the cultivation and the industrial uses of *C. sativa* plants; so it is auspicious to apply proper practices that do not cause an increase of this cannabinoid. Moreover, in the perspective of using hemp inflorescences as a novel food, the possibility of using proper agronomic practices to obtain a specific chemical profile can strongly contribute to enhance the potential nutritional value of this matrix.

## 4. Conclusions

Considering that the food application of hemp inflorescences is a debated topic, this study can contribute to further underline the potential use of this matrix as a food or food ingredient, since several metabolites important from a nutritional and biological point of view were measured. Of course, these kinds of studies, based on chemical characterization, also have to be supported by safety, toxicity, and biological studies to further improve the total eligibility of hemp inflorescences as a novel food. The here reported results suggest the importance to choose the proper cultivation conditions to obtain products with a determined chemical composition, thus offering the possibility to have potential foods or food ingredients with a variable profile based on specific applications. The here reported investigation that involves chemical and agronomic aspects aims to propose a methodological approach that could be useful for future applications in this sector and also for the design of the experimental phase.

## Figures and Tables

**Table 1:** Cannabinoid concentrations of the 2017 and 2018 samples reported for each phosphorus (P) and nitrogen (N) fertilization combination at two harvesting stages. Analyses were carried out in triplicate and expressed as % (*w*/*w*, g for 100 g of dried inflorescence) ± SD.

Harvesting Period	Fertilization	Δ9-THC % *w*/*w*	CBD % *w*/*w*	Δ9-THCA % *w*/*w*	CBDA % *w*/*w*	CBG % *w*/*w*	CBC % *w*/*w*
2017 year							
Stage A	P0 N0 I	0.08 ± 0.01 ^a^	1.74 ± 0.14 ^a^	/	0.77 ± 0.10 ^a^	/	0.08 ± 0.01 ^a^
	P0 N150 I	0.06 ± 0.01	1.58 ± 0.09	/	0.60 ± 0.14	/	0.07 ± 0.01
	P90 N0 I	0.06 ± 0.01	1.54 ± 0.07 ^a^	/	0.84 ± 0.03 ^a^	/	0.06 ± 0.01
	P90 N150 I	0.07 ± 0.01	1.71 ± 0.17 ^a^	/	0.64 ± 0.04	/	0.08 ± 0.01 ^a^
Stage B	P0 N0 I	0.05 ± 0.01	0.83 ± 0.01	/	0.52 ± 0.01	/	0.05 ± 0.01
	P0 N150 I	0.06 ± 0.01	1.28 ± 0.20	/	0.67 ± 0.05	/	0.06 ± 0.01
	P90 N0 I	0.05 ± 0.01	1.03 ± 0.08	/	0.71 ± 0.03	/	0.05 ± 0.01
	P90 N150 I	0.05 ± 0.01	0.97 ± 0.03	/	0.66 ± 0.03	/	0.05 ± 0.01
2018 year							
Stage A	P0 N0 D	0.08 ± 0.01	0.94 ± 0.04 ^a^	0.07 ± 0.01 ^a,b^	0.47 ± 0.02 ^a,b^	0.07 ± 0.01 ^b^	0.15 ± 0.04 ^a^
	P0 N0 I	0.09 ± 0.01 ^a^	0.60 ± 0.10 ^a,b^	/	0.27 ± 0.03	/^a^	0.19 ± 0.01 ^a^
	P0 N75 D	0.06 ± 0.01	0.70 ± 0.10 ^a^	/	0.39 ± 0.02 ^a,b^	0.04 ± 0.01 ^b^	0.12 ± 0.01 ^a,b^
	P0 N75 I	0.08 ± 0.01 ^a^	0.76 ± 0.02	/	0.29 ± 0.03 ^a^	0.07 ± 0.01 ^a^	0.19 ± 0.01 ^a^
	P0 N150 D	0.06 ± 0.01	1.30 ± 0.02 ^b^	/	0.39 ± 0.03 ^a,b^	0.05 ± 0.01 ^a^	0.12 ± 0.01 ^a,b^
	P0 N150 I	0.07 ± 0.02	0.87 ± 0.02 ^a^	/	0.16 ± 0.01 ^a^	0.07 ± 0.01 ^a^	0.19 ± 0.01 ^a^
	P45 N0 D	0.08 ± 0.01	1.30 ± 0.20 ^b^	0.08 ± 0.01 ^a,b^	0.43 ± 0.01 ^a^	0.05 ± 0.01 ^a^	0.12 ± 0.01 ^a,b^
	P45 N0 I	0.09 ± 0.01	0.45 ± 0.01 ^a^	/	0.45 ± 0.01 ^a^	0.07 ± 0.01 ^a^	0.20 ± 0.01 ^a^
	P45 N75 D	0.06 ± 0.01	0.60 ± 0.10 ^a^	0.07 ± 0.01 ^a,b^	0.34 ± 0.05 ^b^	0.04 ± 0.01 ^a^	0.11 ± 0.01 ^a,b^
	P45 N75 I	0.08 ± 0.01	0.60 ± 0.10 ^a^	/	0.10 ± 0.01 ^a^	0.06 ± 0.02	0.19 ± 0.01 ^a^
	P45 N150 D	0.06 ± 0.01 ^b^	1.00 ± 0.10 ^a,b^	0.08 ± 0.01 ^a,b^	0.17 ± 0.01 ^a,b^	0.05 ± 0.01 ^a^	0.12 ± 0.01 ^a,b^
	P45 N 150 I	0.09 ± 0.01	0.80 ± 0.04 ^a^	/	0.48 ± 0.04	0.07 ± 0.01 ^a^	0.20 ± 0.01 ^a^
	P90 N0 D	0.07 ± 0.01	0.98 ± 0.01 ^a^	/ ^b^	0.46 ± 0.03 ^a,b^	/ ^b^	0.12 ± 0.01 ^a,b^
	P90 N0 I	0.11 ± 0.03 ^a^	1.30 ± 0.50	0.13 ± 0.01 ^a^	0.35 ± 0.03	0.08 ± 0.01 ^a^	0.22 ± 0.03 ^a^
	P90 N75 D	0.06 ± 0.01 ^b^	0.55 ± 0.05 ^a,b^	0.07 ± 0.01 ^b^	0.55 ± 0.04 ^a,b^	/ ^b^	0.11 ± 0.01 ^b^
	P90 N75 I	0.09 ± 0.01	0.93 ± 0.01 ^a^	/	0.31 ± 0.01 ^a^	0.07 ± 0.01 ^a^	0.20 ± 0.01 ^a^
	P90 N150 D	0.07 ± 0.01	0.93 ± 0.08	/	0.18 ± 0.02 ^a,b^	0.05 ± 0.01 ^a,b^	0.12 ± 0.01 ^a,b^
	P90 N150 I	0.09 ± 0.01	0.92 ± 0.07	/	0.46 ± 0.03 ^a^	0.08 ± 0.01 ^a^	0.20 ± 0.01 ^a^
Stage B	P0 N0 D	0.07 ± 0.01	1.72 ± 0.09	/	0.30 ± 0.01	0.05 ± 0.01	0.07 ± 0.01
	P0 N0 I	0.06 ± 0.01	1.60 ± 0.27	/	0.30 ± 0.01	0.04 ± 0.01	0.09 ± 0.03
	P0 N75 D	0.07 ± 0.01	1.40 ± 0.01 ^b^	/	0.72 ± 0.01 ^b^	0.05 ± 0.01 ^b^	0.07 ± 0.01 ^b^
	P0 N75 I	0.05 ± 0.01	0.80 ± 0.10	/	0.36 ± 0.02	/	/
	P0 N150 D	0.08 ± 0.01	1.38 ± 0.06	/	0.74 ± 0.01 ^b^	/ ^b^	0.07 ± 0.01 ^b^
	P0 N150 I	0.07 ± 0.01	1.30 ± 0.10	/	0.33 ± 0.03	0.04 ± 0.01	0.13 ± 0.01
	P45 N0 D	0.08 ± 0.01	1.43 ± 0.01	/	0.67 ± 0.02	/	0.07 ± 0.01 ^b^
	P45 N0 I	0.08 ± 0.01	1.60 ± 0.27	/	0.66 ± 0.03	/	0.12 ± 0.01
	P45 N75 D	0.06 ± 0.01	1.33 ± 0.05 ^b^	/	0.32 ± 0.01 ^b^	/ ^b^	0.07 ± 0.01 ^b^
	P45 N75 I	0.08 ± 0.01	1.10 ± 0.10	/	0.55 ± 0.01	0.04 ± 0.01	0.12 ± 0.01
	P45 N150 D	0.06 ± 0.01	1.36 ± 0.01 ^b^	/	0.30 ± 0.01 ^b^	/ ^b^	0.07 ± 0.01 ^b^
	P45 N 150 I	0.08 ± 0.01	1.25 ± 0.01	/	0.56 ± 0.03	0.04 ± 0.01	0.13 ± 0.01
	P90 N0 D	0.08 ± 0.01	1.67 ± 0.01 ^b^	/	0.29 ± 0.01 ^b^	/	0.08 ± 0.01 ^b^
	P90 N0 I	0.08 ± 0.01	1.13 ± 0.05	/	0.39 ± 0.01	/	0.11 ± 0.01
	P90 N75 D	0.07 ± 0.01	0.99 ± 0.13 ^b^	0.05 ± 0.01 ^b^	0.77 ± 0.02	/	0.09 ± 0.04
	P90 N75 I	0.08 ± 0.01	1.35 ± 0.13	/	0.80 ± 0.10	/	0.12 ± 0.01
	P90 N150 D	0.07 ± 0.01	1.01 ± 0.06	0.05 ± 0.01 ^b^	0.53 ± 0.07 ^b^	/	0.06 ± 0.01 ^b^
	P90 N150 I	0.07 ± 0.01	1.01 ± 0.05	/	0.72 ± 0.03	/	0.12 ± 0.01

^a^ *p* < 0.05, significantly different than the corresponding sample harvested in stage B, ^b^ *p* < 0.05, significantly different than the corresponding irrigated sample (one-way ANOVA, followed by Turkey HSD Test). Stage A = female flowering stage, stage B = seed maturity stage, I = irrigation, D = no irrigation.

**Table 2:** Total phenolic content (TPC, mg kg^−1^ of fresh inflorescences ± SD) and antioxidant activity (inhibition rate I% ± SD) by spectrophotometric analysis of the 2017 and 2018 samples reported for each phosphorus (P) and nitrogen (N) fertilization combination at two harvesting stages. Analyses were carried out in triplicate.

Harvesting Period	Fertilization	TPC (mg kg^−1^ Fresh Hemp)	DPPH (EC_50_ in mg Fresh Hemp mL^−1^)	ABTS (mg TE g^−1^ Fresh Hemp)
2017 year				
Stage A	P0 N0 I	3788.15 ± 198.83 ^a^	0.84 ± 0.32 ^a^	0.75 ± 0.34
	P0 N150 I	3911.21 ± 158.83 ^a^	0.82 ± 0.46 ^a^	0.75 ± 0.42
	P90 N0 I	3859.71 ± 311.84 ^a^	0.82 ± 0.14	0.75 ± 0.45
	P90 N150 I	3607.90 ± 278.35	0.88 ± 0.52	0.75 ± 0.46
Stage B	P0 N0 I	3201.61 ± 175.29	0.93 ± 0.01	0.75 ± 0.14
	P0 N150 I	3515.79 ± 88.44	0.97 ± 0.29	0.75 ± 0.19
	P90 N0 I	3031.07 ± 198.78	0.95 ± 0.37	0.75 ± 0.27
	P90 N150 I	3351.50 ± 33.45	0.96 ± 0.62	0.75 ± 0.24
2018 year				
Stage A	P0 N0 D	1686.54 ± 88.95 ^a^	0.72 ± 0.26 ^a^	0.76 ± 0.38
	P0 N0 I	1811.72 ± 190.37 ^a^	0.74 ± 0.57	0.75 ± 0.43
	P0 N75 D	2958.75 ± 455.53 ^a,b^	0.90 ± 0.52	0.76 ± 0.17
	P0 N75 I	1712.82 ± 4.04 ^a^	0.68 ± 0.30	0.74 ± 0.29
	P0 N150 D	1610.49 ± 10.75 ^a^	0.70 ± 0.23	0.75 ± 0.01
	P0 N 150 I	1605.26 ± 7.18 ^a^	0.72 ± 0.19	0.76 ± 0.66
	P45 N0 D	1650.77 ± 206.59 ^a^	0.75 ± 0.36	0.76 ± 0.15
	P45 N0 I	1766.50 ± 36.08	0.74 ± 0.43	0.74 ± 0.49
	P45 N75 D	1884.70 ± 103.77 ^a^	0.70 ± 0.20	0.75 ± 0.11
	P45 N75 I	1752.48 ± 62.43 ^a^	0.75 ± 0.18	0.76 ± 0.44
	P45 N150 D	1806.23 ± 3.89 ^a,b^	0.69 ± 0.34	0.76 ± 0.51
	P45 N 150 I	1496.75 ± 5.26 ^a^	0.79 ± 0.14	0.76 ± 0.58
	P90 N0 D	2806.45 ± 198.60 ^a,b^	0.78 ± 0.11	0.76 ± 0.50
	P90 N0 I	1603.93 ± 8.84 ^a^	0.71 ± 0.30	0.75 ± 0.16
	P90 N75 D	1598.98 ± 1.20 ^a^	0.70 ± 0.17	0.76 ± 0.38
	P90 N75 I	1758.90 ± 118.94 ^a^	0.86 ± 0.16	0.76 ± 0.18
	P90 N150 D	1478.74 ± 52.86 ^a,b^	0.77 ± 0.15	0.75 ± 0.31
	P90 N150 I	2074.89 ± 144.55	0.87 ± 0.01	0.76 ± 0.65
Stage B	P0 N0 D	2114.52 ± 206.76	0.83 ± 0.06	0.74 ± 0.35
	P0 N0 I	2517.61 ± 158.69	0.79 ± 0.51	0.76 ± 0.66
	P0 N75 D	1827.67 ± 183.84 ^b^	0.64 ± 0.60	0.76 ± 0.01
	P0 N75 I	2441.43 ± 69.45	0.83 ± 0.04	0.74 ± 0.25
	P0 N150 D	1235.93 ± 37.86 ^b^	0.69 ± 0.42	0.76 ± 0.33
	P0 N150 I	1408.64 ± 37.68	0.91 ± 0.45	0.75 ± 0.19
	P45 N0 D	2176.90 ± 192.05	0.77 ± 0.03	0.76 ± 0.09
	P45 N0 I	2339.09 ± 490.98	0.86 ± 0.24	0.75 ± 0.17
	P45 N75 D	2514.91 ± 7.71 ^b^	0.77 ± 0.46	0.76 ± 0.22
	P45 N75 I	2480.07 ± 0.35	0.83 ± 0.14	0.75 ± 0.07
	P45 N150 D	2089.19 ± 25.88 ^b^	0.71 ± 0.38	0.75 ± 0.18
	P45 N 150 I	2535.18 ± 42.93	0.79 ± 0.29	0.76 ± 0.01
	P90 N0 D	2183.52 ± 104.90 ^b^	0.68 ± 0.17	0.75 ± 0.17
	P90 N0 I	2618.61 ± 10.21	0.86 ± 0.43	0.75 ± 0.22
	P90 N75 D	2283.75 ± 67.69	0.66 ± 0.24	0.75 ± 0.22
	P90 N75 I	2462.76 ± 234.60	0.91 ± 0.67	0.76 ± 0.21
	P90 N150 D	2335.18 ± 60.61	0.67 ± 0.05	0.75 ± 0.02
	P90 N150 I	2177.69 ± 191.41	0.91 ± 0.23	0.76 ± 0.48

^a^ *p* < 0.05, significantly different than the corresponding sample harvested in stage B, ^b^ *p* < 0.05, significantly different than the corresponding irrigated sample (one-way ANOVA, followed by Turkey HSD Test). Stage A = female flowering stage, stage B = seed maturity stage, I = irrigation, D = no irrigation.

**Table 3:** Biomass yield, inflorescences yield, and inflorescences/biomass ratio (inf/bio) of the 2017 and 2018 samples reported for each phosphorus (P) and nitrogen (N) fertilization combination at two harvesting stages. Measurements were carried out in triplicate.

Harvesting Period	Fertilization	Biomass (kg ha^−1^)	Inflorescences (kg ha^−1^)	Inf/Bio
2017 year				
Stage A	P0 N0 I	4540.11 ± 346.75 ^a^	616.61 ± 34.16 ^a^	0.14 ± 0.01 ^a^
	P0 N150 I	6476.78 ± 293.35 ^a^	840.66 ± 38.91 ^a^	0.13 ± 0.01 ^a^
	P90 N0 I	4932.39 ± 380.13 ^a^	616.09 ± 60.50 ^a^	0.12 ± 0.01 ^a^
	P90 N150 I	6717.79 ± 173.38 ^a^	862.28 ± 45.66 ^a^	0.13 ± 0.01 ^a^
Stage B	P0 N0 I	5618.85 ± 258.66	967.38 ± 112.69	0.17 ± 0.01
	P0 N150 I	8115.37 ± 133.85	1281.79 ± 89.10	0.16 ± 0.01
	P90 N0 I	5967.57 ± 288.33	1032.14 ± 79.09	0.17 ± 0.01
	P90 N150 I	8429.39 ± 478.14	1446.22 ± 99.63	0.17 ± 0.01
2018 year				
Stage A	P0 N0 D	1567.65 ± 161.07 ^b^	217.15 ± 27.87 ^a,b^	0.14 ± 0.01 ^a^
	P0 N0 I	4839.72 ± 346.76 ^a^	679.69 ± 24.19 ^a^	0.14 ± 0.01 ^a^
	P0 N75 D	1245.49 ± 80.32 ^a,b^	170.37 ± 4.56 ^a,b^	0.14 ± 0.01 ^a^
	P0 N75 I	5636.54 ± 682.71 ^a^	776.25 ± 72.03 ^a^	0.14 ± 0.01 ^a^
	P0 N150 D	1061.00 ± 106.68 ^a,b^	143.97 ± 19.53 ^a,b^	0.14 ± 0.01 ^a^
	P0 N 150 I	6877.93 ± 221.06 ^a^	976.34 ± 56.00 ^a^	0.14 ± 0.01 ^a^
	P45 N0 D	1533.95 ± 49.27 ^a,b^	210.27 ± 18.05 ^a,b^	0.14 ± 0.01 ^a^
	P45 N0 I	5215.94 ± 579.36 ^a^	718.69 ± 100.28 ^a^	0.14 ± 0.01 ^a^
	P45 N75 D	1373.71 ± 131.17 ^b^	174.38 ± 29.37 ^a,b^	0.13 ± 0.01 ^a^
	P45 N75 I	5990.38 ± 199.68 ^a^	824.92 ± 25.75 ^a^	0.14 ± 0.01 ^a^
	P45 N150 D	1085.79 ± 12.56 ^b^	143.32 ± 17.78 ^a,b^	0.13 ± 0.01 ^a^
	P45 N 150 I	7181.05 ± 380.83 ^a^	993.08 ± 47.33 ^a^	0.14 ± 0.01 ^a^
	P90 N0 D	1534.55 ± 93.93 ^a,b^	209.62 ± 20.90 ^a,b^	0.14 ± 0.01 ^a^
	P90 N0 I	5356.79 ± 475.80 ^a^	700.44 ± 50.03 ^a^	0.13 ± 0.01 ^a^
	P90 N75 D	1292.04 ± 187.33 ^b^	177.43 ± 14.05 ^a,b^	0.14 ± 0.01 ^a^
	P90 N75 I	6046.85 ± 366.29 ^a^	807.35 ± 68.69 ^a^	0.13 ± 0.01 ^a^
	P90 N150 D	1154.10 ± 231.08 ^b^	170.97 ± 18.02 ^a,b^	0.15 ± 0.01 ^a^
	P90 N150 I	7125.98 ± 313.94 ^a^	984.88 ± 51.93 ^a^	0.14 ± 0.01 ^a^
Stage B	P0 N0 D	1883.45 ± 154.60 ^b^	341.47 ± 37.53 ^b^	0.18 ± 0.01
	P0 N0 I	6265.84 ± 422.05	1100.76 ± 41.47	0.18 ± 0.01
	P0 N75 D	1548.50 ± 88.45 ^b^	261.39 ± 31.92 ^b^	0.17 ± 0.01
	P0 N75 I	7213.44 ± 565.81	1284.05 ± 96.02	0.18 ± 0.0
	P0 N150 D	1363.78 ± 78.17 ^b^	226.66 ± 29.39 ^b^	0.17 ± 0.01
	P0 N150 I	8377.37 ± 223.66	1510.85 ± 85.60	0.18 ± 0.01
	P45 N0 D	1945.90 ± 142.73 ^b^	329.57 ± 50.37 ^b^	0.17 ± 0.01
	P45 N0 I	6489.49 ± 468.28	1180.99 ± 177.02	0.18 ± 0.01
	P45 N75 D	1558.84 ± 92.52 ^b^	285.11 ± 17.12 ^b^	0.18 ± 0.01
	P45 N75 I	7451.66 ± 364.56	1308.29 ± 90.31	0.18 ± 0.01
	P45 N150 D	1204.93 ± 144.89 ^b^	233.02 ± 24.49 ^b^	0.19 ± 0.01
	P45 N 150 I	8655.74 ± 514.37	1629.76 ± 102.02	0.19 ± 0.01
	P90 N0 D	1945.84 ± 168.74 ^b^	334.18 ± 54.75 ^b^	0.17 ± 0.01
	P90 N0 I	6528.28 ± 410.66	1215.83 ± 135.20	0.19 ± 0.01
	P90 N75 D	1536.66 ± 129.23 ^b^	274.72 ± 28.83 ^b^	0.18 ± 0.01
	P90 N75 I	7408.61 ± 105.87	1363.41 ± 60.00	0.18 ± 0.01
	P90 N150 D	1177.53 ± 109.00 ^b^	250.68 ± 31.20 ^b^	0.21 ± 0.01 ^b^
	P90 N150 I	8635.76 ± 396.31	1590.19 ± 97.10	0.18 ± 0.01

^a^ *p* < 0.05, significantly different than the corresponding sample harvested in stage B, ^b^ *p* < 0.05, significantly different than the corresponding irrigated sample (one-way ANOVA, followed by Turkey HSD Test). Stage A = female flowering stage, stage B = seed maturity stage, I = irrigation, D = no irrigation.

## Data Availability

Not applicable.

## Supplementary Materials

The following supporting information can be downloaded at: https://www.mdpi.com/article/10.3390/foods11223658/s1, Figure S1. Schematic diagram explaining the practices of nitrogen (N) and phosphorus (P) fertilizations and irrigation applied for the cultivation of Ferimon cultivar. Subscript numbers refer to the kg of fertilizer applied for ha. Table S1. Means of minimum (T. min) and maximum (T. max.) air temperatures, total rainfall and potential evapo-transpiration (ET0) throughout the periods of study at 10-days intervals from March to September in 2017 and 2018 cropping season. Table S2. Quantified metabolites in Bligh-Dyer hydroalcoholic extracts of Ferimon inflorescences, with relative ^1^H NMR signals (ppm) used for quantification. Table S3. NMR quantitative results of amino acids measured in Bligh-Dyer hydroalcoholic extracts of Ferimon inflorescences in 2017. Table S4. NMR quantitative results of organic acids, sugars and other molecules measured in Bligh-Dyer hydroalcoholic extracts of Ferimon inflorescences in 2017. Table S5. NMR quantitative results of amino acids measured in Bligh-Dyer hydroalcoholic extracts of Ferimon inflorescences in 2018. Table S6. NMR quantitative results of organic acids, sugars and other molecules measured in Bligh-Dyer hydroalcoholic extracts of Ferimon inflorescences in 2018.
