# Supplementary material for: Industrial Hemp (Cannabis sativa L.) Inflorescences as Novel Food: The Effect of Different Agronomical Practices on Chemical Profile
Source: Foods. 2022 Nov 16;11(22):3658. doi: 10.3390/foods11223658 (PMC9689267; doi:10.3390/foods11223658)
Supplement: Supplementary file 1 [file foods-11-03658-s001.zip › foods-1991165-supplementary.pdf]

SUPPLEMENTARY MATERIAL

Industrial Hemp (*Cannabis sativa* L.) Inflorescences as Novel Food: The Effect of Different Agronomical Practices on Chemical Profile

Mattia Spano [1], Giacomo Di Matteo [1], Cinzia Ingallina [1], Anatoly Petrovich Sobolev [2,*], Anna Maria Giusti [3], Giuliana Vinci [4], Silvia Cammarone [1], Carola Tortora [1], Lara Lamelza [1], Sabrina Antonia Prencipe [4], Laura Gobbi [4], Bruno Botta [1], Federico Marini [5], Enio Campiglia [6] and Luisa Mannina [1]

[1] Department of Chemistry and Technology of Drugs, Sapienza University of Rome, Piazzale Aldo Moro 5, 00185 Rome, Italy
[2] Magnetic Resonance Laboratory "Segre-Capitani", Institute for Biological Systems, CNR, Via Salaria Km 29.300, 00015 Monterotondo, Italy
[3] Department of Experimental Medicine, Sapienza University of Rome, Piazzale Aldo Moro 5, 00185 Rome, Italy
[4] Department of Management, Sapienza University of Rome, Via del Castro Laurenziano 9, 00161 Rome, Italy
[5] Department of Chemistry, Sapienza University of Rome, Piazzale Aldo Moro 5, 00185 Rome, Italy
[6] Department of Agricultural and Forest Sciences, University of Tuscia, Via San Camillo de Lellis Snc, 01100 Viterbo, Italy
* Correspondence: anatoly.sobolev@cnr.it

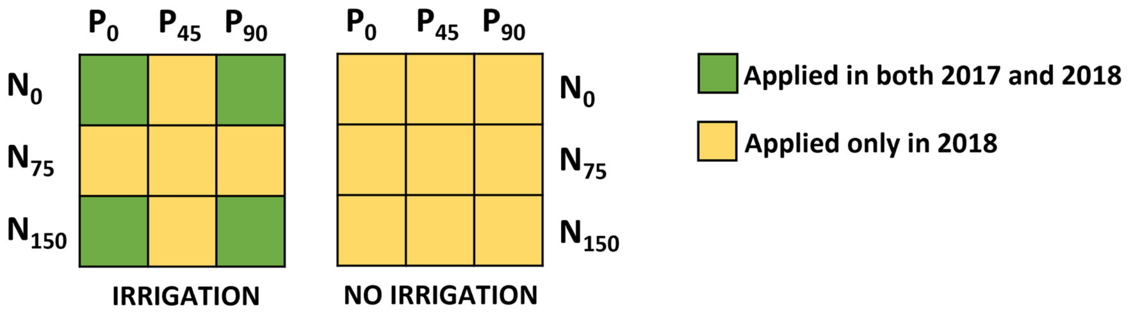

Figure S1. Schematic diagram explaining the practices of nitrogen (N) and phosphorus (P) fertilizations and irrigation applied for the cultivation of Ferimon cultivar. Subscript numbers refer to the kg of fertilizer applied for ha.

Table S1. Means of minimum (T. min) and maximum (T. max.) air temperatures, total rainfall and potential evapotranspiration (ET0) throughout the periods of study at 10-days intervals from March to September in 2017 and 2018 cropping season.

	2017 year				2018 year			
	T. Min. [°C]	T. Max. [°C]	Rainfall [mm]	ET0 [mm]	T. Min. [°C]	T.Max. [°C]	Rainfall [mm]	ET0 [mm]
March	4.2	15.6	41	20	2.6	12.9	61	14
	4.1	17.2	0	23	5.8	14.4	85	18
	5.4	19.9	0	30	4.2	14.2	43	31
April	6.9	20.4	4	28	6.4	18.6	33	29
	6.1	19.9	2	33	10.7	21.8	7	35
	4.8	18.4	23	32	9.5	24.9	0	42
May	8.3	21.5	17	35	12.1	20.6	91	25
	11.9	25.4	0	45	10.7	21.5	49	37
	12.0	27.0	1	56	14.9	26.6	10	47
June	13.2	28.7	0	52	13.4	27.2	6	46
	15.7	31.6	0	57	16.5	28.1	4	56
	17.0	31.3	16	50	14.7	27.5	4	53
July	16.4	32.7	0	54	17.3	30.7	0	57
	16.9	32.4	0	57	16.6	32.0	27	57
	16.7	32.0	48	53	18.4	32.8	6	65
August	19.9	37.8	0	56	20.4	33.6	25	54
	17.2	32.1	25	49	18.4	31.3	55	48
	17.1	33.2	2	54	16.8	29.4	29	50
September	13.8	25.8	77	31	15.4	27.8	36	38
	12.2	23.6	16	26	16.4	28.5	36	32
	10.5	23.3	4	25	12.6	26.3	1	39
	5.4	19.9	0	30	5.4	19.9	0	30

Table S2. Quantified metabolites in Bligh-Dyer hydroalcoholic extracts of Ferimon inflorescences, with relative ^{1}H NMR signals (ppm) used for quantification.

**Compound**	**^{1}H chemical shift, ppm**	**Compound**	**^{1}H chemical shift, ppm**
Isoleucine	1.02	Choline	3.21
Valine	1.05	Myo-inositol	3.30
Threonine	1.34	Fructose	4.04
Alanine	1.49	Malate	4.30
Proline	2.00	β-Glucose	4.66
Citrate	2.55	α-Glucose	5.24
GABA	2.30	Sucrose	5.42
Succinate	2.41	Phenylalanine	7.43
Glutamine	2.46	Tryptophan	7.53
Aspartate	2.83	Formate	8.46
Asparagine	2.89	Trigonelline	9.12

Table S3. NMR quantitative results of amino acids measured in Bligh-Dyer hydroalcoholic extracts of Ferimon inflorescences in 2017.

**Sample**	**Metabolite, mg/100 g**									
	Ile	Val	Thr	Ala	Pro	GABA	Gln	Asp	Asn	Phe	Trp
P0 N0 I A	0.91	0.53	0.43	1.77	4.10	5.80	3.56	2.62	10.51	0.52	1.28
P0 N150 I A	2.27	0.61	1.16	2.25	27.22	5.85	5.20	3.27	42.80	1.35	0.88
P90 N0 I A	0.94	0.45	0.59	2.66	6.61	7.54	4.63	2.59	15.73	0.45	1.50
P90 N150 I A	1.54	0.45	1.08	2.29	13.42	5.31	4.14	3.73	35.60	1.18	1.96
P0 N0 I B	1.10	0.31	1.07	2.49	3.58	8.82	24.90	6.17	95.32	0.57	0.56
P0 N150 I B	0.98	0.18	0.69	1.88	6.05	6.76	17.28	4.36	36.25	0.85	1.51
P90 N0 I B	0.84	0.21	0.59	1.55	1.73	5.41	11.27	3.76	35.48	0.78	0.79
P90 N150 I B	1.10	0.31	0.94	1.90	2.26	6.25	27.07	6.06	79.88	0.81	1.25

Table S4. NMR quantitative results of organic acids, sugars and other molecules measured in Bligh-Dyer hydroalcoholic extracts of Ferimon inflorescences in 2017.

**Sample**	**Metabolite, mg/100 g**								
	Formate	Citrate	Succinate	Malate	Glucose	Myo-Inositol	Fructose	Sucrose	Trigonelline	Choline
P0 N0 I A	0.39	15.57	1.11	11.70	108.66	20.28	66.65	60.75	2.89	3.58
P0 N150 I A	0.35	15.45	1.63	21.10	93.83	17.37	56.47	35.04	3.40	3.95
P90 N0 I A	0.36	13.27	1.45	11.91	151.67	27.24	113.17	111.87	3.89	3.59
P90 N150 I A	0.31	13.19	1.30	16.32	105.00	16.98	57.05	32.70	3.54	3.58
P0 N0 I B	0.59	17.47	7.80	11.05	134.66	9.56	102.80	54.23	5.84	4.15
P0 N150 I B	0.44	18.99	5.41	6.90	153.08	7.30	116.03	77.64	6.24	3.22
P90 N0 I B	0.40	12.39	3.53	7.04	115.00	6.77	72.97	20.76	3.34	3.43
P90 N150 I B	0.81	11.46	8.48	7.18	110.66	5.88	68.37	20.66	5.44	3.80

Table S5. NMR quantitative results of amino acids measured in Bligh-Dyer hydroalcoholic extracts of Ferimon inflorescences in 2018.

**Sample**	**Metabolite, mg/100 g**									
	Ile	Val	Thr	Ala	Pro	GABA	Gln	Asp	Asn	Phe	Trp
P0N0 I A	1.06	5.06	2.25	15.43	12.72	34.00	8.95	1.75	29.65	1.84	10.55
P0N75 I A	2.25	3.42	2.11	13.79	2.71	33.88	6.78	0.97	14.60	3.13	5.06
P0N150 I A	1.76	4.94	2.39	18.60	11.79	38.51	4.34	0.95	36.21	1.91	9.72
P45N0 I A	3.04	5.20	2.73	19.30	11.57	43.67	10.19	0.87	25.66	3.09	6.93
P45N75 I A	2.36	4.18	2.06	14.77	6.74	36.29	10.27	1.00	28.44	2.86	5.95
P45N150 I A	2.53	3.99	4.94	20.40	5.31	35.35	4.73	1.31	20.47	3.74	4.94
P90N0 I A	1.68	3.73	1.23	20.39	6.07	41.03	6.65	0.87	33.50	3.65	3.34
P90N75 I A	1.55	4.38	1.88	18.91	12.38	43.67	4.30	1.57	35.99	2.22	8.28

P90N150 I A	1.24	3.44	1.64	14.33	11.61	31.34	7.11	1.58	32.92	3.17	6.43
P0N0 D A	2.56	2.23	2.52	28.99	25.76	40.92	3.88	1.88	28.06	2.61	5.21
P0N75 D A	1.03	5.15	2.42	33.74	35.63	48.83	9.65	2.32	63.87	3.86	4.78
P0N150 D A	1.23	5.12	1.98	28.92	44.51	47.26	6.64	2.45	60.47	3.38	12.59
P45N0 D A	1.20	6.10	2.19	43.72	64.51	49.82	8.89	4.49	54.66	4.93	3.21
P45N75 D A	1.41	6.42	1.10	36.17	51.25	41.35	9.73	2.37	56.32	4.73	3.82
P45N150 D A	2.11	8.47	1.90	45.29	68.18	49.90	10.39	2.29	82.56	5.16	4.73
P90N0 D A	1.69	8.00	1.32	106.46	55.83	54.34	5.01	3.20	81.76	4.95	2.64
P90N75 D A	7.87	7.27	3.14	44.21	75.33	52.71	6.74	4.66	77.51	3.94	5.63
P90N150 D A	3.10	6.32	2.42	36.01	52.65	48.33	8.68	1.67	50.31	4.52	7.07
P0N0 I B	1.33	3.49	1.26	12.31	12.58	29.95	23.73	3.70	81.20	1.57	13.45
P0N75 I B	2.79	4.93	2.56	24.46	8.81	38.90	19.81	4.29	90.30	1.85	9.39
P0N150 I B	3.14	5.23	3.22	21.54	19.68	43.44	29.22	4.51	115.57	2.41	8.26
P45N0 I B	1.56	3.84	2.00	13.16	15.41	27.85	21.48	3.34	77.87	1.64	13.24
P45N75 I B	2.23	4.69	2.61	28.14	15.26	39.33	19.84	4.58	133.77	2.37	10.65
P45N150 I B	3.06	5.32	2.50	27.80	18.23	36.04	23.59	5.34	132.71	2.38	8.55
P90N0 I B	2.06	3.38	2.66	9.85	7.04	28.87	27.24	4.80	110.30	11.38	21.60
P90N75 I B	1.29	3.29	2.13	15.76	28.51	29.51	16.50	2.75	62.60	2.47	7.42
P90N150 I B	2.45	5.38	3.15	25.86	33.14	39.83	21.37	11.21	107.91	2.72	7.60
P0N0 D B	1.25	6.36	3.16	28.54	27.49	45.59	33.40	5.50	169.13	5.46	8.55
P0N75 D B	1.90	4.70	1.01	30.25	16.61	44.09	10.64	5.98	101.57	2.71	8.41
P0N150 D B	2.59	5.69	3.31	32.71	21.38	44.74	30.59	7.14	130.90	3.90	8.61
P45N0 D B	0.88	5.79	1.87	33.72	18.56	46.57	23.14	5.85	140.49	3.15	12.19
P45N75 D B	2.15	4.58	3.22	26.30	22.11	38.94	18.60	4.84	105.65	1.66	10.90
P45N150 D B	2.23	5.96	5.09	38.12	26.09	49.62	19.79	6.84	126.75	4.40	10.14
P90N0 D B	0.98	4.89	2.57	35.03	18.93	50.22	31.22	4.91	127.12	3.86	9.81
P90N75 D B	2.64	6.36	4.50	34.91	24.91	56.78	23.23	8.97	163.64	3.46	10.76
P90N150 D B	2.92	5.63	4.76	35.54	23.91	49.83	20.28	6.89	119.42	3.13	7.23

Table S6. NMR quantitative results of organic acids, sugars and other molecules measured in Bligh-Dyer hydroalcoholic extracts of Ferimon inflorescences in 2018.

**Sample**	**Metabolite, mg/100 g**									
	Formate	Citrate	Succinate	Malate	Glucose	Myo-Inositol	Fructose	Sucrose	Trigonelline	Choline
P0N0 I A	0.66	63.61	19.66	7.24	107.48	46.94	168.07	348.98	7.86	10.71
P0N75 I A	0.95	62.57	16.95	8.54	97.27	55.41	127.77	206.71	6.55	11.94
P0N150 I A	0.77	54.55	15.03	8.82	107.26	52.83	122.16	298.47	7.88	12.67
P45N0 I A	1.05	63.25	21.76	8.03	144.84	62.32	186.13	315.82	8.93	14.14
P45N75 I A	1.01	56.96	19.07	8.22	124.16	64.65	177.84	328.38	8.53	11.86
P45N150 I A	0.92	33.97	19.18	9.04	147.61	60.01	156.50	243.31	7.73	13.02
P90N0 I A	0.90	74.74	29.85	9.35	250.97	57.99	179.13	429.41	8.67	14.97
P90N75 I A	0.98	92.01	25.01	14.86	111.33	45.54	130.58	334.38	10.42	12.71
P90N150 I A	0.73	86.37	25.10	16.79	162.18	44.76	237.83	526.06	8.40	12.40
P0N0 D A	1.15	72.76	20.99	29.58	218.86	76.70	206.13	337.38	12.01	15.60
P0N75 D A	1.13	105.50	31.25	21.23	265.74	83.21	316.89	676.75	14.47	20.24
P0N150 D A	1.52	108.10	25.94	19.19	273.83	67.47	275.64	615.77	15.79	19.13
P45N0 D A	1.67	109.56	30.59	40.22	323.01	87.68	353.30	683.32	19.91	24.16
P45N75 D A	1.07	81.76	28.13	32.99	269.48	84.89	268.24	513.47	18.18	17.54
P45N150 D A	1.65	89.13	29.59	29.34	324.08	94.88	285.07	671.47	21.47	23.21
P90N0 D A	1.51	89.42	28.51	21.20	299.84	25.23	213.02	562.53	22.60	22.34
P90N75 D A	1.51	106.38	18.96	54.29	335.92	84.15	260.20	619.39	26.10	23.50
P90N150 D A	1.48	71.04	29.73	31.08	302.74	82.96	265.54	426.48	20.72	19.44
P0N0 I B	1.04	92.50	16.08	20.46	152.61	23.85	131.29	191.63	6.91	9.68
P0N75 I B	1.57	47.74	14.08	11.69	194.87	32.90	107.19	93.27	8.67	12.47
P0N150 I B	1.44	72.69	15.65	16.77	291.70	30.78	194.03	159.26	9.67	13.32
P45N0 I B	1.24	72.46	14.60	13.28	168.65	28.53	168.86	228.90	7.06	10.12
P45N75 I B	1.40	63.42	14.32	17.83	188.02	40.48	156.37	187.28	8.68	14.53
P45N150 I B	1.62	58.11	13.58	21.81	188.39	28.41	170.35	208.58	9.91	13.47
P90N0 I B	1.18	96.71	22.69	18.07	236.19	24.89	166.70	173.95	9.56	11.39
P90N75 I B	0.80	37.60	10.77	7.76	209.28	25.07	126.90	138.27	6.74	9.07
P90N150 I B	2.39	38.58	9.34	11.53	308.44	33.27	179.24	38.31	10.23	13.64
P0N0 D B	2.06	133.16	24.50	50.72	245.56	31.60	231.55	402.76	12.87	15.37
P0N75 D B	1.82	100.49	22.11	26.10	208.29	32.00	150.85	276.55	12.02	14.23
P0N150 D B	2.63	124.86	24.24	71.81	226.62	36.90	214.82	421.60	13.86	15.85
P45N0 D B	1.93	114.09	25.30	40.60	190.71	29.95	90.11	431.87	12.64	15.88
P45N75 D B	1.65	101.11	22.09	39.98	160.17	25.65	143.73	287.45	11.83	12.77
P45N150 D B	1.66	112.43	26.41	56.63	225.90	32.39	173.04	418.94	15.08	14.93
P90N0 D B	1.61	111.20	22.30	51.71	214.76	27.42	191.79	384.04	11.78	15.46
P90N75 D B	1.83	123.52	27.92	54.06	214.06	32.39	198.26	364.43	14.64	15.36
P90N150 D B	2.26	117.83	19.21	52.62	234.13	36.34	182.91	303.56	13.01	17.70
